# Unraveling the pros and cons of various in vitro methodologies for ruminant nutrition: a review

**DOI:** 10.1093/tas/txac130

**Published:** 2022-09-15

**Authors:** James R Vinyard, Antonio P Faciola

**Affiliations:** Department of Animal Sciences, University of Florida, Gainesville, FL 32611, USA; Department of Animal Sciences, University of Florida, Gainesville, FL 32611, USA

**Keywords:** batch culture, cell culture, continuous culture, microbial ecology, nutrient degradation, pure culture

## Abstract

To decrease the time and cost of experiments as well as the use of animals in nutrition research, in vitro methodologies have become more commonplace in the field of ruminant nutrition. Therefore, the objectives of this review are 1) to describe the development of different in vitro methodologies, 2) to discuss the application, utilization, and advantages of in vitro methodologies, 3) to discuss shortcomings of in vitro methodologies, and 4) to describe the potential developments that may be able to improve in vitro methods. Having been used for decades, some in vitro methodologies such as pure, batch, and continuous cultures have been very well documented and utilized to investigate a wide array of different aspects of nutrition, including the effects of different dietary compositions, individual fermentation end products, and impacts on the microbiome of the rumen. However, both batch and pure cultures can result in a build-up of end products that may inhibit fermentation, as they culture ruminal contents or defined strains of bacteria, respectfully. Continuous culture; however, allows for the removal of end products but, similar to pure and batch cultures, is applicable only to ruminal fermentation and cannot provide information regarding intestinal digestion and bioavailability. This information for in vitro can only be provided using an assay designed for total tract digestibility, which is the three-step procedure (**TSP**). The TSP may be improved by coupling it with cell culture to investigate the absorption of nutrients in both the ruminal and intestinal phases of the methodology; however, the TSP needs further development to investigate all nutrients and the methodologies available for cell culture are still relatively new to ruminant nutrition. Therefore, while in vitro methodologies provide useful data in the field of ruminant nutrition without the continuous use of animals, there is still much work to be done to improve the methodologies to further apply them.

## INTRODUCTION

In vitro methodologies have been used in animal nutrition research for decades to decrease the use of animals and experimental time and cost. Due to its lower cost, in vitro methodologies are commonly used for preliminary testing, prior to large in vivo trials. The use of in vitro also helps researchers to focus on the three R’s of animal research: reduce, refine, and replace (Curzer et al., 2016). When in vitro methodologies are used, they allow researchers to reduce both the number of animals used and their total use, as they only require the collection of inoculum rather than using them to complete lengthy digestibility or production trials. They refine their use, as tissues are either collected from animals that are being harvested for meat otherwise or ruminal cannulae are placed in a small number of animals rather than using a large group for in vivo study. In some instances, such as pure and cell culture, the use of animals is eventually even replaced, as the unit of study becomes refined strains of bacteria or cells. However, there are many different needs that can be met by in vitro methods, whether that be ruminal degradation, impacts on microbial communities and ruminal fermentation like batch or continuous cultures (Arce-Cordero et al., 2022), estimating total tract digestibility using three-step procedures (Calsamiglia and Stern, 1995), or determining specific microbial affects using pure bacterial cultures or epithelial cell cultures (Zhan et al., 2020).

Due to the wide variety of the investigatory capabilities of in vitro methodologies, there are various experimental designs that can achieve adequate statistical power, including completely randomized, completely randomized block, and Latin square; along with the factorial or split-plot arrangement of treatments. These different designs were reviewed in depth by Hristov et al. (2019) and thus will not be discussed in detail here. As a whole, in vitro is used in instances where the use of in vivo is inappropriate, due to risk to animals, costly, or as a preliminary experiment. For instance, in vitro can be used to test levels of toxin exposure (Dai et al., 2019; Kent-Dennis et al., 2020) to animals that may be of a level that would cause illness or death in vivo, without risking an animal’s wellbeing. In vitro is also a very useful tool when investigating the specific physiological mechanisms of a microbial species or for a pilot study to determine the efficacy of a new rumen-protected product.

While they are well documented and some have been evaluated meta-analytically, to our knowledge, there is no in-depth review of in vitro methodology that discusses the techniques and applications for pure, batch, continuous, and cell cultures and the three-step methods for determination of digestibility. Thus, to fulfill the gap in the literature, the objectives of this review are to 1) describe the development of different in vitro methodologies, 2) discuss their application, utilization, and advantages, 3) discuss their shortcomings, and 4) describe potential developments that may be able to improve these methodologies.

## PURE CULTURE

### Development

The use of pure culture to investigate the characteristics of bacterial species has been used for decades (Bryant and Burkey, 1953). Pure culture consists of growing a specific strain of bacteria in its ideal media, typically in a culture tube, and applying the treatment of interest to determine the change in fermentation profile specific to that bacterial strain. Their initial use in ruminant nutrition was to determine the nutritional requirements of specific microorganisms and the end-products that they synthesize from nutrients (Bryant, 1959; Hungate et al., 1964). Further work was conducted to develop methods to also determine the enzymatic activity from microbial enzymes within a pure culture to further understand the action of microorganisms therein (Joyner and Baldwin, 1966), which has shaped the trajectory of pure culture since.

### Utilization

As pure culture does not include interactions between groups of microorganisms, it is ideal for investigating mechanisms of microbial synthesis, particularly for intermediates that may be consumed or otherwise altered by other microorganisms in a more dynamic system. For instance, Dewanckele et al. (2020) utilized pure culture to investigate 28 different ruminal bacterial species and their production of *trans*-10 fatty acid intermediates of C18 polyunsaturated fatty acids to determine specific bacterial species to be targeted to reduce the production of trans-10 intermediates to potentially prevent milk fat depression. They were able to determine that only one species was capable of producing *trans*-10 fatty acid intermediates under simulated conditions. Other species (i.e., *Megasphaera elsdenii* and *Propionibacterium acnes*) had previously been identified to also produce *trans*-10 fatty acids in vitro (Kim et al., 2002), but those intermediates had been quickly converted to hydroxy fatty acids rather than remaining as *trans*-10 intermediates. Thus, Dewanckele et al. (2020) were able to determine that *Cutibacterium acnes* is an important producer of a *trans*-10 fatty acids from polyunsaturated fatty acids that may lead to further inhibition of milk fat synthesis and lead to milk fat depression (Harvatine et al., 2009).

Pure culture can also be used to study the impact of external factors on specific microorganisms. Dai et al. (2020) examined the impact of exposing ruminal bacteria to lipopolysaccharide (LPS), which is suspected to be a contributing factor to ruminal acidosis. They were able to find that LPS increased the growth rate of bacterial species that fermented sugars and produced lactate, which could further exacerbate conditions of ruminal acidosis. Thus, they furthered the understanding of mechanisms involved in ruminal acidosis by evaluating the impact of LPS on bacteria. Similar work has been done to further understand the impact of toxins from plants and fungi on ruminal microorganisms (Loh et al., 2020). The evaluations of toxin exposure in microorganisms are particularly of importance in ruminal metabolism, as some ruminal microorganisms detoxify certain molecules, rendering them inert, allowing the use of feeds that may be considered toxic in other species (Dai et al., 2020; Loh et al., 2020). A pure culture is used to evaluate the impact of toxins due to its specificity to individual species of microorganisms and its ability to be used without potentially endangering an animal due to toxin exposure.

### Shortcomings

The biggest shortcoming of pure culture, and all other culture methods, is the limited scope of the experiments that are able to be conducted. Most bacterial species found in nature are not able to be cultured in a laboratory setting (Stewart, 2012; Vartoukian et al., 2010). The bacteria found in the rumen are no different in that regard as there are many species that are known to be present in the rumen, but have not been able to be cultured and studied in vitro. Other culture methodologies may be able to avoid this by using an adaptation period to create a more stable environment or fresh ruminal content samples in short-term incubations (i.e., batch culture) to limit native microbial loss. However, pure culture is unable to do so as it requires defined media and isolated cultures of microorganisms.

The environment created within pure culture experiments is also a limitation of the scope of the experiments that can be carried out. Even in a diverse defined consortium of bacteria, there are still missing interactions with other microorganisms or their natural products that may impact the growth of the microorganisms of interest (Vartoukian et al., 2010). The use of a defined media rather than the environment of the rumen could also impact growth and kinetics as the presence of an array of nutrients that are being produced and actively removed in the rumen cause fluctuations in pH that may not be able to be replicated in pure culture, depending on what is being cultured. Fluctuations of pH have been demonstrated to reduce or halt the growth of some strains of bacteria and cause others to flourish, thus a stable pH in pure culture may limit the natural growth patterns that could be observed (Russell and Dombrowski, 1980).

## BATCH CULTURE

### Development

The development of the batch culture incubation methodology for the in vitro fermentation of feed ingredients was initially reported by Tilley and Terry (1963) and further updated by Goering and Van Soest (1970). The history of batch culture was extensively reviewed by Yáñez-Ruiz et al. (2016), but in brief, these methodologies require the collection of ruminal fluid, diluting the fluid with buffer, and incubating it in closed bottles with the substrate of interest. Following incubation, the contents are filtered and analyzed to determine the digestion that occurred.

These methodologies have been used to quickly estimate the degradation of nutrients and nutritional quality of feed ingredients. Some methodologies utilize the same principles as the original methodologies but take advantage of more recent technologies. Holden (1999) compared the use of serum bottles against the Ankom DAISY^II^ (Ankom Technology Corp., Macedon, NY) with the DAISY^II^ being tested using bags containing all the same feed or with different feeds. The DAISY^II^ is an incubation cabinet that contains four rotating jars containing buffered ruminal fluid that are used to incubate many samples that have been weighed into nylon bags at the same time. Holden (1999) compared a different methodology for ten different feeds using a version of the original Tilley and Terry (1963) methodology adapted for use with the DAISY^II^ and found that DM degradability was not impacted by the methodologies used or the presence of different diets within the same fermentation vessel, allowing for the authors to evaluate ten different feeds for a fraction of the time, cost, and labor involved in in vivo research.

### Utilization

While simple, batch culture has a wide variety of analytes that it can be used for, including gas production, fermentation end products, nutrient degradation, and microbial communities. As batch culture is a fully closed system, gas production measurements are simple and can measure through changes in pressure in the bottle and concentrations of different gasses therein (Theodorou et al., 1994). We will not discuss gas production via batch culture in great detail as Yáñez-Ruiz et al. (2016) have provided an in-depth review on the subject. However, in brief, in vitro gas production has been measured in different methodologies since the 1940s (Quin, 1943) and has been updated several times (Menke and Steingass, 1988; Theodorou et al., 1994), but the initial method largely ignores the extent and rate of fermentation. Eventually, updates to the methodology continued to develop the automated measurements that are common today (Cornou et al., 2013; Muetzel and Tavendale, 2014).

The current batch culture methodology allows for evaluation of the quality of fermentation and extent of nutrient degradation throughout incubation. This allows for the evaluation of fermentation profiles and end-products (organic acids, NH_3_-N, pH, and microbial ecology) as well as the degradation of nutrients. With that, the methodology has been developed to analyze different fractions of carbohydrates and proteins, particularly to determine fractions of rumen undegraded protein (RUP) and rumen degraded protein (RDP) and the undegradable fractions of fiber (uNDF). A distinct advantage of batch culture is the ability to test a large number of treatments at one time. For example, Saleem et al. (2022) were able to investigate the fermentation profile and gas production for 55 different types of wheat samples that were each processed in three different ways for a total of 165 different samples in duplicate incubations. An equivalent study in vivo, or even using a different culture method, could have taken years to achieve the same results that were obtained in a matter of weeks using batch culture.

### Shortcomings

While beneficial in its ease of analysis, batch culture is not without flaws in its design. The closed system allows for the buildup of both fermentation end-products and pressure due to gas produced within the fermentation vessel, which has the potential to alter results due to an inhibition of both the rate and the extent fermentation (Tagliapietra et al., 2010) and impact on end-product production (Jouany and Lassalas, 2002). The buildup of organic acids and gasses within the fermentation vessel in batch culture may lead to a decrease in overall pH, which in turn could impact microbial ecology and the fermentation therein (Russell and Hespell, 1981), thus batch culture vessels must be vented to release gas in longer incubations and buffers are used to limit the impact of end-products.

Due to this, the results of batch culture studies cannot necessarily be directly applied to make assumptions of responses in vivo as the removal of end-products may elicit different results.However, this is true of any in vitro methodology and batch culture is a very useful tool when first evaluating treatments, feeds, or additives as it provides a quick response to elucidate the potential impact a treatment could have on fermentation. While the results of the primary evaluation may not be the same result as observed in vivo, they would be able to show the direction in which further evaluation should be headed.

## CONTINUOUS CULTURE

### Development

Continuous culture fermentation (CC) was originally described by Hobson (1965a, 1965b) and involves the maintenance of an in vitro culture of ruminal fluid, over a longer period of time, as compared to some other in vitro methods. As described in a meta-analysis by Hristov et al. (2012), the three main advantages of CC are low experimental cost, the ability to test treatments with sufficient statistical power in a short amount of time, and the ability to examine the effects of treatments that we would not be able to investigate in vivo (i.e., high levels of toxins or extreme acidosis challenges). All three of these are the advantages of in vitro experiments, regardless of the method; however, CC has one distinct advantage over the other types of in vitro methodologies discussed here and that is the removal of fermentation end products allowing for a longer stable fermentation.

**Figure 1.**
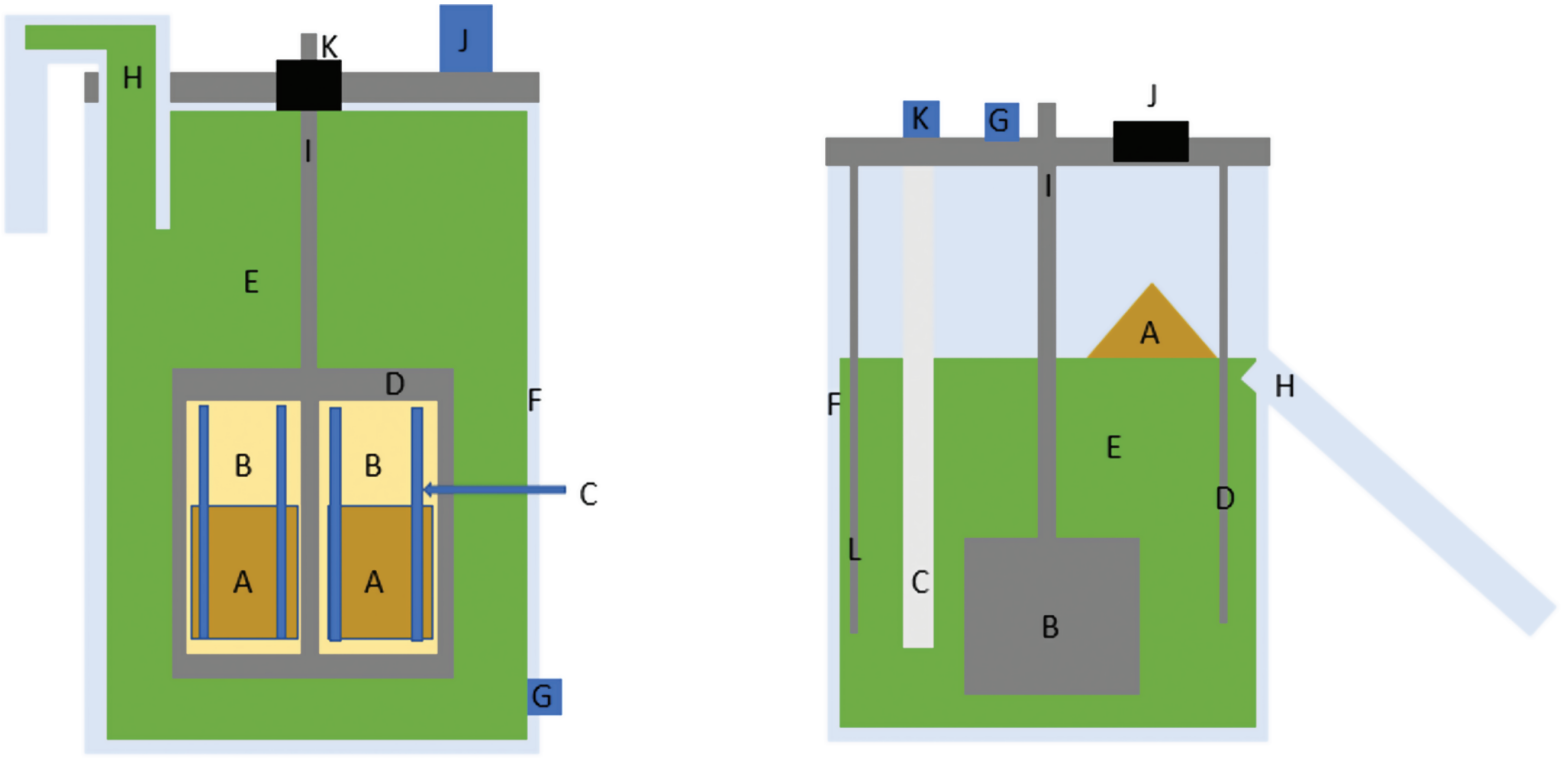
a). Schematic diagram of the rumen simulation technique (RUSITEC) system adapted from Czerkawski and Breckenridge (1977). A, feed within nylon bag; B, porous nylon bag; C, rigid tube used to support bags; D, perforated container; E, ruminal fluid; F, fermentation vessel; G, inlet for infusion of artificial saliva; H, outlet for digesta removal via overflow; I, drive shaft for rotation; J, sampling port; K, airtight rubber seal. b). Schematic diagram of the dual-flow continuous culture system first utilized by Monteiro et al. (2022). A, feed added directly to the ruminal content; B, agitator/mixer; C, filter for constant removal of liquid fraction; D, temperature sensor; E, ruminal content; F, fermentation vessel; G, inlet for infusion of artificial saliva; H, outlet for digesta removal via overflow; I, drive shaft; J, opening for addition of feed/sampling port; K, connection to peristaltic pump for liquid removal; L, heater.

The types of CC include the single-flow (SFCC) and dual-flow (DFCC) methodologies. The flows of which, refer to the outflow of effluent from the system. In SFCC, the outflow of effluent comes from one single exit (either via overflow of vessel contents or pumped out at a controlled rate) and is a mixture of the solid and liquid fractions of the effluent. One type of SFCC is the rumen simulation technique (RUSITEC). First described by Czerkawski and Breckenridge (1977), RUSITEC uses nylon bags of feed within a CC of ruminal fluid that is maintained with constant agitation, the inflow of artificial saliva, and outflow due to overflow; illustrated in Figure 1a, the original RUSITEC design employs the use of an air-tight vessel, making measurement of gas production possible (Martínez et al., 2009).

The use DFCC was first described by Hoover et al. (1976) and the outflow is separated into solid and liquid fractions in which the outflow via overflow is the solid fraction and the filtered, the pumped outflow is the liquid fraction; illustrated in Figure 1b. Thus, the response is more representative of what would be observed in vivo than SFCC. A meta-analysis conducted by Brandao et al. (2020) investigated the relationship between responses reported in vivo using the omasal sampling technique (OST; Huhtanen et al., 1997; Ahvenjärvi et al., 2000) and the DFCC system. The OST is well-established technique for the evaluation of ruminal degradability. Thus, the data collected in OST experiments should be comparable to those collected in those using DFCC. Brandao et al. (2020) utilized data from 155 articles (97 DFCC and 58 OST) to investigate the correlation between results from DFCC and OST. They found that most response variables were similar between DFCC and OST, and when they were different, it was due to a difference in the intercept. Thus, indicating that the patterns reported in the data were similar, but were of different magnitudes between DFCC and OST meaning that although the exact values reported may be different, the changes reported therein will be similar. In the same vain, in another meta-analysis conducted by Brandao and Faciola (2019), investigated the differences observed across in different DFCC studies. The authors investigated the impact of dietary composition, specifically CP and NDF, and the amount of feed given each day on the end products of microbial fermentation. They found that the estimates of ruminal degradation, concentrations of VFA, and N metabolism were similar between studies using the DFCC system.

### Utilization

Methodology for CC has been well documented; however, it has not, to our knowledge, been extensively reviewed. Both SFCC and DFCC have been used to study changes in the carbohydrate (Benedeti et al., 2015; Carro et al., 2009; Ravelo et al., 2021; Vallimont et al., 2004), lipid (Brandao et al., 2018b; Castillejos et al., 2005), protein (Amaral et al., 2016; Brandao et al., 2018a; Paula et al., 2017), vitamin (Arce-Cordero et al., 2022) and mineral (Agustinho et al., 2022; Arce-Cordero et al., 2021) fractions of diets, as well as the inclusion of feed additives like direct fed microbials (Miller-Webster et al., 2002; Monteiro et al., 2022) and exogenous enzymes (Bennett et al., 2021), methane production (Eun et al., 2004; Martínez et al., 2009), or exposure of the ruminal environment to toxins (Dai et al., 2019). As demonstrated by Brandao et al. (2020) and Brandao and Faciola (2019), continuous culture provides meaningful insight into the ruminal fermentation that would be observed in vivo, but for a fraction of the time and cost of an in vivo trial. This is due to evidence indicating that the microbial communities in CC experiments stabilize after four days, rather than two or more weeks in vivo (Salfer et al., 2018). Different CC trials have been used to investigate ruminal fermentation and nutrient degradation, the flow and metabolism of N, microbial metabolism of nutrients (primarily N), changes in microbial ecology, and the production of gasses. Due to the small volume of the fermentation vessel (less than 2 L) and ease of application via artificial saliva, labeled ^15^N is commonly used as a marker to determine microbial uptake and utilization of N within the system.

In the meta-analysis conducted by Hristov et al. (2012), they compared CC (both single- and dual-flow), RUSITEC, and in vivo degradability trials to determine the differences in variability across the different study types for ruminal fermentation and nutrient degradation data. They reported that CC had greater variation in its data than RUSITEC, which was more variable than in vivo studies. It is important to note that the data regarding CC did not separate SFCC and DFCC data, which could be a source of variation, and used a small selection of in vivo data (366 cows from only three Universities) compared to 30 years of CC data (1074 different treatments) which could explain the lack of variation in the in vivo data. This is evidenced in the meta-analyses by Brandao and Faciola (2019) and Brandao et al. (2020) that demonstrated the consistencies across DFCC methodologies and their results as compared to OST. Along with all in vitro methods, CC is constantly being improved. Wenner et al. (2021) altered the original DFCC methodology by changing the shape of the fermenter vessel to be more rounded, increasing the diameter of the impeller, improving the motor for the impeller, and adding a better filtration system for liquid flow; all of which decreased variability when compared to DFCC studies conducted using the original system.

### Shortcomings

Beyond the already discussed shortcomings of in vitro methodologies, CC is not without further limitations. The first of which is the costs of building the system, maintaining the system, and the operation of the experiments. One of the appeals of in vitro work is that it is less expensive than in vivo experiments that would investigate the same parameters (Hristov et al., 2012). However, the startup and upkeep of CC systems is daunting and for a system that may not be used consistently, spending the resources on in vivo trials or multiple other in vitro methodologies may be more cost-effective. This may be the reason that there are so few CC systems in use, currently. However, a CC system that is used regularly and continuously can be reliable and maintenance costs can be spread over a number of experiments, thus creating a cost-effective use of the system.

Another detriment of the CC methodology is the inability to culture protozoa at the same levels reported in vivo, which could potentially alter ruminal fermentation. The initial methodology that developed DFCC (Hoover et al., 1976) was originally investigated in an effort to improve the retention of protozoa in CC, but reported lower protozoal counts than the initial RUSITEC method by Czerkawski and Breckenridge (1977). Hristov et al. (2012) reported that protozoal counts were higher for non-RUSITEC CC methods; however, they were still lower than those reported in vivo, including some that were not detectable. Some of those studies even reported that protozoal populations were not detectable within the culture. The dramatic decrease in protozoal populations is thought to be due to the combined effects of the outflow of solids from the CC system and the low replication rate for ruminal protozoa resulting in the failure to maintain the population in culture (Martínez et al., 2010). However, it has been demonstrated that total counts and concentrations of bacteria, fungi, and methanogenic archaea decrease during CC fermentation as well when compared to their concentrations at inoculation (Mateos et al., 2017). Thus, indicating that while CC may not maintain microbial populations at the same levels as those reported in vivo (Martínez et al., 2010; Mateos et al., 2017) they are still able to provide meaningful results with similar differences as what would be observed in vivo (Brandao et al. 2020).

## TOTAL TRACT IN VITRO DIGESTIBILITY

### Development

While the estimation of ruminal digestion is relatively simple as ruminal content is readily accessible, the collection of inoculum for in vitro digestion from the abomasum or small intestine becomes more complex as the collection of contents from either is challenging. Thus, the development of in vitro methodologies using chemicals was essential to providing a reliable methodology for in vitro estimation of total tract digestibility. Initially, Calsamiglia and Stern (1995) developed the three-step procedure (TSP) for the determination of total tract digestibility of protein, as other available methodology, like the mobile bag technique (Hvelplund, 1985) that utilizes duodenal cannulated animals, or acid detergent insoluble nitrogen (Goering et al., 1972), were highly variable. Thus, Calsamiglia and Stern (1995) developed a consistent assay that was validated against samples obtained from an in vivo intestinal digestibility study.

The initial TSP was developed for the determination of protein digestibility and estimation of RDP and RUP fractions of feeds (Calsamiglia and Stern, 1995). It consisted of a 16 h in situ ruminal fermentation step, a 1 h acid/pepsin incubation, and a 24 h incubation in a buffered pancreatin solution. The pancreatin used is a powdered extract containing pancreatic enzymes derived from the porcine pancreas. That methodology has since been updated several times to increase its validation and scope. The first update came from Gargallo et al. (2006) to include the use of the Ankom Daisy^II^ and then Ross et al. (2013) further updated the method to eliminate the use of bags that could potentially inhibit microbial attachment to feed particles and provide amounts of individual pancreatic enzymes to use rather than use pancreatin, which can be variable between batches. Further updates were reported by Vinyard et al. (2021) to adapt the methodology to determine lipid digestibility by adding bile and calcium to the intestinal digestion step of the TSP.

### Utilization

The TSP is varied in its design across the literature and can combine different aspects of in vitro and in situ. The initial methodology (Calsamiglia and Stern, 1995) used in situ for ruminal incubation and followed it with in vitro for the abomasal and intestinal steps, whereas the other methodologies (Gargallo et al., 2006; Ross et al., 2013; Vinyard et al., 2021) use only in vitro. The methodologies described by Calsamiglia and Stern (1995) and Gargallo et al. (2006) utilize bags to contain feeds, which have been demonstrated to limit microbial attachment (Schlau et al., 2021), but also limit the scope of the diets that can be investigated (i.e. cannot contain fine particles or liquids). Both Ross et al. (2013) and Vinyard et al. (2021) forego the use of bags and utilize the analysis of digestion end products to determine digestibility.

### Shortcomings

One of the major concerns with the TSP, as discussed by Ross et al. (2013) is the varied use of enzymes and enzyme mixtures between methods. Pancreatin, which is an extract of pancreatic enzymes, varies between extraction batches and the company producing the product. This variation could reduce the repeatability of the method as discussed by Ross et al. (2013) in the reasoning for using individual enzymes rather than pancreatin. Inconsistencies between enzyme concentrations reported in vivo and those used in in vitro methodologies can lead to a difference in the magnitude of the observed results between the two. However, in vitro methodologies are not utilized to provide exact results as to what would be observed with the same application in vivo and can only be used in order to estimate the effects that would be seen. For instance, if a decrease in digestibility was observed in vitro, a similar decrease would be expected in vivo, but the magnitude of that decrease may be different between the two studies. Thus, in vitro methodology is typically only used to collect preliminary data to predict an outcome in vivo, rather than to obtain definitive results that are directly applicable to animal production.

As with any previously discussed batch culture style experiments, the end products of digestion, fermentation in particular, can inhibit the extent of digestion. In the TSP, the added inhibition comes in the final step in which enzymes for digestion may not be released from the substrate. End products of digestion, as well as the different digestive agents, can potentially inhibit digestion in batch culture setup due to the continued binding of substrates that prevent enzymes from being recycled and prevent nutrients from being digested further. For example bile, which was utilized by Ross et al. (2013) and Vinyard et al. (2021), when included in solution at concentrations greater than 5 g/L, it can become inhibitory to lipid digestion as it prevents lipase action by preventing its attachment to fatty acids.

As with any of the previously discussed methodologies, the TSP does not take the absorption of nutrients into account. The only difference is that the TSP doesn’t account for absorption in both the ruminal and intestinal phases of the method, whereas the prior methodologies only have the ruminal portion. This may lead to either the underestimation of digestibility as mentioned previously or the overestimation of the nutrient availability as it is assumed that all of the digested material will be absorbed. Thus, if in vitro methodology as a whole, but specifically the TSP, is to become more reliable absorption of end products will need to become part of the methodology to paint an accurate picture.

## CELL CULTURE

### Development

The commonality in the shortcomings of all previously described in vitro methodology is the lack of investigation into physiological impacts. In more recent years, technology has advanced for the ability to grow cultures of epithelial cells from digestive epithelium in the lab. With this advancement, those cultured cells can be used to investigate the impact that a diet would have on the epithelial cells themselves and also the absorption and bioavailability of the digested nutrients.

While relatively novel to ruminant nutrition, cell culture has been utilized in studies investigating human metabolism for decades (Vincent et al., 1985). In short, an immortal line of human colonic epithelial cells (Caco-2) are grown in a media in which the cells can proliferate and be utilized for experimentation. The immortal line grows quickly and is theoretically more hardy than the primary cells. This quick growth and hardiness allow for simpler upkeep in the lab as compared to primary cells. The Caco-2 methodology has been utilized in the study of human metabolism for decades to investigate the impact of different nutrients, pharmaceuticals, and other factors on cellular health, nutrient absorption and utilization, and bioavailability in the human intestinal epithelium. However, this technology is just beginning to be utilized in the field of ruminant nutrition and, to our knowledge, only a few studies have utilized this methodology thus far.

### Utilization

The utilization of cell culture in ruminant nutrition is of interest primarily for two different areas of epithelium: ruminal and intestinal. Kent-Dennis et al. (2020) examined the use of rumen epithelial cells (REC) to investigate the impact of LPS on the rumen epithelium and inflammation therein. To do so, they collected epithelial tissue from the rumens of Holstein bull calves and heifers that were already being harvested and isolated the epithelial cells for culture. Once cultured, the cells in the first experiment were exposed to different dose levels of LPS. The authors found that LPS dose did not alter cell viability, but they found increased expression of toll-like receptors and pro-inflammatory cytokines. In the second experiment, the cells were exposed to LPS at two different doses for different amounts of time; including removing LPS from the media. This resulted in similar observations to the first experiment, but they found that the removal of LPS resulted in a return to baseline levels of expression in REC, indicating that REC can recover following exposure to LPS. Both of the experiments conducted by Kent-Dennis et al. (2020) would have required exposure of animals to high levels of LPS followed by euthanasia for tissue collection, leading away from the three R’s of animal research by not reducing the number of animals used or replacing their use with other available methods. Thus, cell culture could provide a viable option for these types of studies, in particular when animals will be harvested for meat anyway.

To further understand the cellular metabolism of VFA, Zhan et al. (2020) isolated epithelial cells from the jejunum of Chinese Holstein calves to create an immortal cell line of bovine intestinal epithelial cells. To do so, they collected epithelium cells from the calves, cultured them in media to allow them to grow, and then inoculated them with a lentivirus that expressed SV40 large T antigen to immortalize them and allow them to continue to proliferate. Once immortalized, they incubated the cells in a medium that contained 20 m*M* of a mixture of VFA. They found that in cells exposed to the VFA media, there was an increase in the expression of genes responsible for VFA transporters, and an increase in uptake of propionate and butyrate. There was also an increase in the expression of genes associated with gluconeogenesis, thus suggesting that exposure to VFA regulates the expression of genes relative to their uptake in the intestinal epithelium. While not entirely impossible, an experiment such as this would have been very difficult and highly invasive to carry out in vivo. Thus, the advances in cell culture methodology will create the opportunity to further study nutrient uptake and regulation within the intestinal epithelium of ruminants.

### Shortcomings

Cell culture methodologies, particularly those with immortal cell lines, are still relatively new in the ruminant nutrition field. As with any new methodology, its implementation requires further examination and repetition to refine the methods. As noted by Zhan et al. (2017), non-immortal cell line culture, like that utilized by Kent-Dennis et al. (2020), is not very efficient for experimental use, as it leads to a high percentage of cell death while attempting to culture the cells. However, immortal cell lines are abnormal in nature due to the cancerous properties that make them immortal; making the non-immortal cells arguably the best cell lines to provide the most accurate response. Thus, leading to the necessity for the improvement of the current methodology to improve both the efficiency of the techniques and the breadth of studies using the methodologies for cell culture.

However, the implementation of cell culture is limited by the methodologies themselves as they require specific equipment and care that may be costly and time-consuming, along with the animals from which the cells are required. However, as with the CC methods, the cost of the equipment and maintenance is part of the methodology and properly trained individuals will be able to conduct experiments using cell culture methodologies to investigate aspects of cellular metabolism that may have not been able to be studied previously, making the cost worthwhile. As for tissue collection from animals, Kent-Dennis et al. (2020) and Zhan et al. (2020) were able to collect tissues from other terminal studies that were being conducted and tissues can also be collected from animals that are being harvested in an abattoir to prevent the death of an animal solely for a small tissue sample.

## CONCLUSIONS

Although they are not without their shortcomings, both individually and as a whole, in vitro methodologies provide a useful tool to researchers wishing to study both specific and broad effects caused by different factors within ruminant nutrition. A wide range of studies has been conducted using pure and batch cultures to quickly investigate the impacts on specific microorganisms and nutrient digestion. Continuous culture has been used as a consistent replacement for in vivo ruminal digestion studies and dual-flow continuous culture has been demonstrated to yield results that are just as meaningful as omasal sampling would yield. While other methodologies only investigate the ruminal aspect of digestion, the three-step procedure allows for the analysis of both abomasal and intestinal digestion, but it warrants further investigation into the bioavailability of nutrients that are digested. Potentially, the bioavailability of those nutrients could be investigated utilizing cell culture methodologies of the intestinal epithelium. While these methodologies are well documented, further investigation and adaptation are needed to address their flaws and potentially combine methodologies to further the use of in vitro methodologies in ruminant nutrition. However, regardless of the in vitro method, the use of in vitro should be solely used for the ranking of treatments and initial investigation into what impacts could potentially be seen in vivo. Thus, the application of any in vitro results to animal performance would be an extrapolation.
